# Practical Observations on Prolapsus of the Rectum

**Published:** 1832-04-01

**Authors:** 


**Practical Observations on Prolapsus of the Rectum.** By *Frederick Salmon*, F.R.C.S. Author of a Practical Essay upon Contraction of the Rectum, Piles, &c. Senior Surgeon of the General Dispensary, Aldersgate Street. Octavo, with Plates, pp. 105. London, 1831.

No maladies are more distressingly annoying to a patient, and more perplexing to a surgeon, than those of the rectum and adjacent parts. The reason of this is abundantly obvious; for, in addition to the ordinary exciting causes of disease, which equally affect the same structures in whatever part of the body they exist, the lower gut and urinary organs are almost constantly exposed to the presence of acrid irritating matters, and are necessarily affected by the various agents by which the body relieves itself of them. These causes add very much to the difficulty of treatment; and seldom are the discriminating intelligence and practical skill of a surgeon more apparent and more beneficial than when directed to the management of the different morbid conditions of these passages. It is not merely by the application of proper topical remedies, or by superior adroitness in operating that he is to expect satisfactory success;—to benefit his patient, as he ought to do, it is necessary that he be a minute and diligent observer of every occurrence, and especially of the different excretions; on the condition of which, both the constitutional health, and the soundness of particular organs of the body, depend.

The importance of this duty is perhaps more directly obvious, and has certainly been more appreciated in the treatment of urinary diseases, which have of late claimed to themselves the searching investigation of some of our best surgeons and physicians; who are at length well satisfied that it is not by torturing their patients with bougies and catheters, or by the indiscriminate use of diuretics, that they can hope to afford relief; but that their practice must be regulated by the state and composition of the urinary secretion;—and how is this state to be ascertained, except the fluid is frequently examined and tested?

The very admirable reflections of Mr. Brodie in his surgical lectures, abundantly demonstrate the importance of such accurate observation; and we wish now strongly to impress on our readers the great utility of attending to the varying conditions of the alimentary excretions, whenever the rectum becomes the seat of disease. The very fact that prolapsus and other morbid states so frequently owe their origin to some cause or another affecting the alvine discharges is a strong argument to urge us to the study; but we shall not enlarge on this topic at present, but proceed to lay before our readers the more important facts connected with the subject of prolapsus ani, or prolapsus recti, as Mr. Salmon more properly names it. To understand the nature of this malady, attention must be given to the anatomy of the lower gut. It sweeps down from the sigmoid flexure of the colon to the anus along the hollow of the sacrum, and is intimately joined to the urethra and neck of the bladder in the male, and to the vagina in the female;—its tissues are the same, as in the other bowels, but the muscular coat is considerably thicker and stronger; and its contractile expelling power is greatly promoted by the action of the powerful levator ani, which is a fleshy funnel, embracing the pelvic viscera, and much contributing to their strength and security. The antagonist to this muscle is the sphincter ani. During health, there is, what may be termed, a mutually-accommodating sympathy between these two, so that when one acts, the other is relaxed, and vice versâ; but in disease, this harmony is disturbed, and much painful distress is thereby occasioned. We shall find that the sphincter is sometimes so rigidly contracted by spasm, as to require division by the knife. Another feature in the general anatomy of the rectum is the abundance and laxity of the cellular substance which surround it, and which is interposed between the different tunics. In some persons this is unusually yielding; and the slightest irritation, either in the bowel itself, or in an adjoining organ, will force the rectum, or at least one or two of its coats to be protruded beyond the sphincter, or, in other words, will occasion a prolapsus. In the early stages it is not a very serious disease, as the gut may be replaced by gentle pressure; and the employment of mild astringent enemas prevents its return; but aggravated cases produce much suffering. Instead of the uniformly soft, smooth, and blood-coloured tumor, as at first, it has become hard, irregular, and dark;—the rugæ of the gut form so many concentric pendulous flaps, which bleed and ulcerate; and the sores thus formed discharge a stinking unhealthy matter; the other symptoms are very well delineated by Mr. Salmon at page 18.

"It now very soon becomes insupportably painful, and, from its magnitude, prevents the passing of the smallest evacuation without acute agony; exclusive of which misery, the patient is often compelled to lie down for an hour, or even two, immediately after the motions are passed, and thus patiently to wait the receding of the swelling. In the end, it descends upon the most trifling exertion, and even spontaneously, so that the sufferer is compelled to retain it within the sphincter, by mechanical assistance." 18.

It is not necessary that we should be detained by any detailed allusion to the ordinary exciting causes of the disease. Mr. Salmon has a very useless chapter, in which he treats of what he calls "constitutional, or those which are connected with some peculiarity of the general health, or the ordinary pursuits and occupations of individuals; and the local, or where the affection occurs as a consequence of some other disease, either in the rectum, or the organs continuous." 10.

All the causes of prolapsus ani are primarily of a local nature; for in what other light are we to view accumulations of hardened fæces, diarrhœa, and hæmorrhoids? although these are ranked by Mr. Salmon under the head of constitutional causes!

We regret to observe a great vagueness of thought and description, and a most negligent want of precision in arranging the materials of the present work; to acquaint his reader that prolapsus ani "may be caused by indigestion; from a general relaxation of the system, or from a want of due attention to diet," cannot fail to be extraordinarily instructive!! He tells us likewise that "another very common source of the disease is sedentary employments, through which the biliary secretion becomes scanty, and a torpid state of the alimentary canal is induced." It is quite apparent that the prolapsus in such cases is attributable to the collection of indurated fæces, or varicose hæmorrhoidal veins, one out of many, of the effects of hepatic disease.

It is an observation worthy of remark, and which we have had very frequent occasion to attend to in practice, that prolapsus ani is especially frequent in those who are, or at any period ever have been, subject to hernia, even though the protrusion is at a distance from the fundament, as when it occurs in the umbilicus or groin. The true explanation of this coincidence we believe to be, an original, and it would seem, even hereditary laxity, or slackness of the cellular tissue and weakness of the muscles at the various abdominal apertures; because in such cases we have remarked the excellent effects of general cold bathing, and of tonic medicines, which tend to brace and give tone to the whole system. If we approved of the term "constitutional cause" as applied to prolapsus of the rectum, we should say that this is the only one with which we are acquainted.

The greater part of the 4th chapter, which professes to treat of the "symptoms" of the disease is unnecessary, as it is equally applicable to any other disease affecting the rectum; and we are inclined to say, that the less that is written on this subject the better; because any lengthened description is apt to draw the attention of the surgeon from the only correct and satisfactory way of knowledge, namely, a visual and manual examination. As long as we have eyes to see and hands to feel with, we cannot easily mistake a prolapsus of the rectum for any other malady;——with as much propriety a surgeon might talk of the symptoms of an ulcer! and forget all the time to describe its appearance, as Mr. Salmon has done, in giving a prosy enumeration of the symptoms which precede, or threaten a prolapsus of the rectum, and neglecting any accurate delineation of the protruded gut.

Without therefore any further ado about "symptoms" we shall proceed to an examination of the only important part of the book, that which explains the treatment of the disease. We do not however intend to occupy four pages by telling our readers that if the general, or constitutional health of our patient is disordered, and debilitated, it is necessary to endeavour to restore and strengthen it; much less shall we give formulas for common aperient pills, and draughts. The only general rule that we shall lay down for practice is that the very mildest aperients are not only much safer, but even more effectual than active or drastic purges; which irritate and vex the rectum. The oleum ricini, and the lac sulphuris are the best; with the occasional use of emollient and astringent enemata.

In the administration of these Mr. Salmon gives the preference to a common elastic bottle and pipe, over any syringe. Under these very simple means, the disease at first is speedily cured; should this however not be the case, and the prolapsus remains exposed, or at least liable to descend very frequently, the surgeon must then ascertain whether there is any stricture of the rectum, by the use of elastic gum bougies. We cannot too forcibly urge the importance of this measure, having so very frequently in practice witnessed the want of success, from the ignorance or neglect of it. The most obstinate and protracted cases have quickly yielded to the cautious use of the bougies, and these must be gradually encreased in size, in order completely to dilate the narrowed part of the rectum. Case 2d very well illustrates the truth of this, and as it, at the same time, faithfully exhibits the progress of a neglected example of prolapsus we shall give it in the words of the patient, as reported in Mr. Salmon's work.

"In September, 1825, I was much annoyed by frequent calls to the water closet. Upon inspection of what passed from me I discovered that it was principally composed of slime and blood. I mentioned this to a medical friend, who prescribed blue pills, and told me that by taking these regularly the dysenteric symptoms would soon vanish. I followed his advice, but derived no benefit. I now began to feel excessive pain at the lower part of the gut at all times, so much so, that I was very uneasy whether sitting or standing. I then applied to the surgeon of the ship I belonged to, who considered it advisable to give me more mercury to produce a soreness of the gums, and he informed me he had no doubt of curing me previous to my being called upon to join my ship in the month of January. He, however, did me no good. During the time I was under his treatment, I was advised to attend strictly to regimen, and was not allowed to eat any animal food. My diet consisted of light puddings and vegetables. Under this regimen I soon became very weak, and much reduced in flesh. The time soon after arrived for me to join my ship, and although little able to bear the fatigues of duty, necessity drove me from the shore, and for a month or six weeks I was without medical advice, and eat and drank just as I had been accustomed, without finding my complaint either the better or the worse for it. On the surgeon joining the ship I placed myself under his care. He explained to me that the frequency of my motions arose from a torpid state of the liver, that bile did not flow regularly, and that a regular course of mercury was the only thing to stimulate the liver to proper action. Mercury was therefore again commenced upon, and shortly after leaving England my mouth became sore, and it was considered right to keep the action up all the way to Bengal. My diet during this time was confined to boiled rice, sago, &c. On the arrival of the ship in India, I had become so emaciated that it was supposed by all my shipmates I could not live long. I was then sent to Calcutta, and placed under the hands of a most respectable physician, who said it was not proper to give me more mercury, and advised me to return to England as soon as possible. I remained at Calcutta six weeks, but derived no benefit from his attendance. The constant desire to go to the stool still remained, and it frequently happened that I could not reach the night stool before the evacuations would pass.

In September, 1826, I embarked, and from that time until my landing in England, in March, 1827, I took no medicine, nor was I under any medical gentleman's care. My appetite was good, but the desire to go to the water closet was as frequent as ever, accompanied by great straining and pain in the rectum. I observed, likewise, that whenever I went to stool the gut used to come down, and I was frequently obliged to put it back with my finger. On my arrival in England I once more put myself under the hands of one of my old shipmates now settled on shore, who after he had attended me about two months, recommended my going to the sea side for the benefit of sea bathing. I therefore went to Plymouth, and soon found myself materially better for the change; I was so far relieved that I slept soundly, was never disturbed in the night by an inclination to go to stool, nor did I pass more than three evacuations during the twenty-four hours, and I was also able to take daily exercise: still blood and slime continued to pass from me at intervals. In October I quitted Plymouth, and once more joined my ship to undertake another voyage to Bengal and China.

I did my duty during the passage tolerably well, being obliged occasionally to lay by, and I returned home at the conclusion of the year 1829, no better, but if any thing worse, than when I left England. During these four years the pain in the rectum has often been very great, and I have frequently felt pains in the loins, and cramps in the legs and thighs." 55.

Four years after the commencement of the disease, the gentleman consulted Mr. Salmon, who treated the case very judiciously and successfully, by suitable internal medicines, and by the use of bougies,—in four months he was restored to perfect health.

From the observations of Mr. Salmon, it appears that, in a good many cases of prolapsed rectum, the cause appears to be, a morbid irritability and spasmodic contraction of the sphincter ani, which thereby becomes a permanent obstacle to the function of the bowel, which cannot be emptied without an unusual degree of effort. The application of steam to the part, and the occasional introduction of a plug or bougie, are generally sufficient to remove this condition; but in some cases, Mr. Salmon has deemed it requisite to divide the sphincter, and even to cut out a triangular portion of it. The scoops which he recommends for the latter purpose appear quite unnecessary; a fine-pointed and sharp scalpel, in the hands of a good surgeon, is by far the best instrument. The dilator introduced to our notice by Mr. S. is a useful instrument while peforming any operations on the rectum. It is provided with a handle at right angles, so that an assistant can retain it in its place; and, at the side, is a wide slit, extending nearly the whole length of the speculum. In dividing the stricture, or in operating for fistula ani, we turn the slit to that part of the bowel which we intend to cut, either from within outwards, or vice versâ; and in the event of hæmorrhage, it is a great advantage to have the rectum kept dilated, as we can so much more easily apply any styptic remedy.

When it is deemed advisable that the prolapsed portion of the rectum should be removed, some surgeons employ the knife, while others recommend the ligature, which, although it produces more pain and irritation, is exempt from the risk of hæmorrhage, often profuse, and occasionally fatal, after cutting operations about the anus. Mr. Salmon assures us that, by the plan which he adopts in removing the protruding gut with the knife, "not only every reasonable apprehension is removed, but the operation altogether is materially simplified," We give his description as related by himself.

"The sufferer being placed in a convenient position, an assistant separating the nates, one or more of the pins, as may be necessary, is to be passed from above downwards, transversely through the basis of the tumour; These penetrating the muscular coat of the bowel, will prevent the return of the intestine after the diseased part is removed. The pain produced by this part of the operation is insignificant.

The prolapsus being thus secured, the operator, with the hook or the forceps, is to lay hold of one of the prominent portions of the tumour, and to draw it gently towards the opposite side; with one stroke of the scissors he is then to remove the part as deep as the line of division between the mucous and muscular coats of the rectum, the latter of which should be left entire, otherwise a permanent difficulty of relieving the bowels will follow the operation. In like manner, all the protruding portions of the prolapsus are in succession to be taken off.

When treating of the morbid anatomy of this disease, I stated that there is in general a portion of the tumour just around the sphincter of a darker colour than the rest. Superficial layers only of this part should be embraced by the forceps and paired off; care being taken not to interfere with the fibres of the internal sphincter, forming the extremity of the rectum.

If any *material* bleeding occurs, it is to be checked by the means generally used for stemming hemorrhage, such as cold or astringent washes. In most cases the flow of blood, which it is better to encourage to a certain degree, will cease spontaneously; but if we are compelled to apply any ligature, it may be done with facility, as while the pins remain in their situation, we have a commanding view of the parts.

It is my custom to leave the pins in their place for an hour or more after the operation, or the cessation of hemorrhage, to permit the blood to coagulate in the extremities of the divided vessels, by which we prevent any recurrence of bleeding after the bowel is restored to its natural situation. Having removed them, the surface of the divided part should be smeared with sweet oil, and the rectum returned within the sphincter in the gentlest possible manner." 10.

We have been induced to extend our observations on the subject of prolapsus ani, much more on account of the importance of the disease, than of the work now before us. It is scarcely entitled to the appellation which the author has given to it of "Practical Observations," having much more the style and character of an inaugural dissertation than of a well-arranged digest of extended experience and of careful observation. The only very useful part of the book is the simple, though apparently successful, modification which he has introduced in the manner of removing the prolapsus by excision. By the employment of the pins, with which he transfixes the swelling, it is prevented from retreating up the gut, and thus the danger of hemorrhage is considerably obviated. Most of the cases at the end of the volume are more serviceable in increasing its bulk, than its utility and reputation. The accompanying plates, illustrating the appearances of prolapsus, are very well lithographed by that excellent artist, Scharf, and printed by Hullmandel.

